# Activity-Related Breathlessness and Physical Activity in Women with Sedentary Behavior: A Cross-Sectional Study

**DOI:** 10.3390/medicina61050880

**Published:** 2025-05-12

**Authors:** Mona Mohamed Taha, Zizi M. Ibrahim, Reem Hamdan Al-Rafdan, Rama Hani Alrehayan

**Affiliations:** Department of Rehabilitation Sciences, College of Health and Rehabilitation Sciences, Princess Nourah bint Abdulrahman University (PNU), Riyadh 11671, Saudi Arabia; zmibrahim@pnu.edu.sa (Z.M.I.);

**Keywords:** dyspnea, breathlessness, female, sedentary lifestyle, women’s health

## Abstract

*Background:* Sedentary behavior is a considerable health risk, which is related to a variety of chronic diseases and a lower quality of life. Breathlessness, or dyspnea, is a significant barrier to physical exercise, especially in sedentary populations. This study aimed to assess the relationship between activity-related dyspnea and physical activity levels in women with sedentary behavior, while also identifying demographic and health factors that influence this association. *Methods:* This study used a cross-sectional design conducted in Saudi Arabia, utilizing an electronic survey for data collection. The participants were Saudi females aged 20–50 years with sedentary behavior, who reported sitting for 6 h or more each day. A self-administered online questionnaire was used to collect data, which examined sociodemographic information, breathlessness (assessed via the Modified Medical Research Council (mMRC) breathlessness scale and the Dyspnea-12 (D-12) questionnaire), and physical activity levels using the Godin Leisure-Time Exercise Questionnaire (GLTEQ). Only healthy volunteers without chronic or respiratory problems were included. *Results:* Among 646 participants, 95.2% reported breathlessness, with Grade 2 (32.2%) being most common. Physical activity levels were low, with 37.36% insufficiently active. Significant differences in dyspnea scores were observed across activity levels (H(2) = 50.43, *p* < 0.0001), with more active individuals reporting less dyspnea. Ordinal logistic regression showed that higher mMRC scores were strongly associated with lower activity (OR = 0.19, 95% CI [0.16, 0.23], *p* < 0.001). Dyspnea-12 physical domain scores also correlated inversely with activity (OR = 0.93, 95% CI [0.90, 0.96], *p* < 0.001), while the emotional domain was not significant. *Conclusions:* The study revealed a significant association between higher breathlessness severity and lower physical activity levels among Saudi women with sedentary behavior. Dyspnea was prevalent, with 95.2% of participants experiencing some degree of breathlessness. Future longitudinal or interventional studies are required to determine the direction of this relationship and explore whether interventions targeting breathlessness management could support increased physical activity or if greater activity itself may help alleviate dyspnea.

## 1. Introduction

Sedentary behavior is any awake behavior that involves sitting, lying, or reclining with an energy expenditure of less than 1.5 metabolic equivalents of task (METs) [1]. Sedentary behavior is distinct from physical inactivity, which refers to an individual who does not engage in moderate-to-vigorous physical activity [2]. Environmental, economic, societal, and technological transformations have contributed to a progressively sedentary lifestyle globally, characterized by practices such as extended television watching and sedentary occupations [3,4,5]. Sedentary behavior is highly prevalent in Saudi Arabia, with 50–95% of the population reporting inadequate physical activity. Approximately half of individuals spend more than five hours per day sitting, and females exhibit significantly higher rates of prolonged sitting compared to males [6]. Rapid economic growth, lifestyle shifts, and climatic extremes that restrict outdoor activities have contributed to this pattern [7].

The health risks of sedentary behavior are extensive. It has been linked to a decreased quality of life, mood disorders, and chronic conditions including type 2 diabetes mellitus, cardiovascular disease, and obesity [8], as well as complications such as sarcopenia, reduced physical activity, and frailty [9].

Breathlessness, commonly known as dyspnea, is a prevalent and distressing symptom characterized by the subjective experience of discomfort in breathing, which can vary in both intensity and sensation [10,11]. This symptom is debilitating, ranking just after pain, and serves as a key indicator of exercise tolerance, quality of life, and mortality across various conditions [12]. Thus, healthcare practitioners are encouraged to routinely assess and document the existence of dyspnea, just as they do for pain [10,13]. Respiratory impairment and dyspnea are believed to contribute to sedentary behavior, as both are associated with lower exercise capacity, reduced training adherence, and a higher risk of physical disability [10,14,15]. Dyspnea is also associated with lower functional capacity and poorer psychological well-being, especially in elderly people [16]. A large population-based study involving middle-aged adults identified elevated body mass index, impaired lung function, and a sedentary lifestyle as key contributors to breathlessness in the general population [17]. Additionally, a community-based study reported that 9–35% of adults suffer from unexplained exertional dyspnea and many people suffering from severe dyspnea do not seek medical care [10,18].

In Saudi Arabia, the research area of sedentary behavior is relatively new in the medical field, and remains underexplored [19]. Although numerous studies have investigated general patterns of physical inactivity, few have specifically addressed the correlation between dyspnea and physical activity levels, especially among women, a demographic disproportionately impacted by sedentary behaviors and underrepresented in scientific research. Examining the factors that lead to physical inactivity and sedentary behavior, along with their associated patterns, is essential. Conditions such as dyspnea may serve both as barriers to and consequences of insufficient physical activity [20,21]. Given the high prevalence of sedentary behavior in Saudi Arabia, particularly among women, and the limited studies exploring its impact, this study aimed to estimate the prevalence of activity-related dyspnea and investigate its association with physical activity levels in sedentary females. Understanding these associations contributes to public health by identifying modifiable barriers to physical activity and informing targeted intervention strategies. We hypothesized that higher levels of dyspnea would be significantly associated with lower physical activity levels in this population.

## 2. Methods

### 2.1. Study Design

The study was a cross-sectional study utilizing an electronic survey. This cross-sectional study followed the Strengthening the Reporting of Observational Studies in Epidemiology (STROBE) checklist [22].

A self-administered online questionnaire was used for the study, which was conducted from May 2023 to May 2024. All participants were given a consent form outlining the study’s aim and role, with the opportunity to reject participation. The trial posed no physical or psychological risks, and participants received no immediate advantages.

### 2.2. Participants

Women aged 20 to 50 years with sedentary behavior in Saudi Arabia were recruited through snowball sampling. They self-reported sedentary behavior, with sitting time recorded as the average hours per day using a single item from the International Physical Activity Questionnaire Short Form [23]. Participants documented the total time spent sitting or reclining during awake hours, encompassing social and recreational activities. Every participant chosen for the study was in good health, able to walk without the need for walking assistance. Exclusion criteria included a history of thoracic surgery, metabolic, osteoarticular, or neuromuscular illnesses, a report of respiratory illness or cold symptoms within the previous three weeks, or smoking. Furthermore, we excluded participants whose body mass index (BMI) was higher than 40 kg/m^2^ or less than 18 kg/m^2^.

Using OpenEpi software 3.01, the required sample size was computed with a 95% confidence level, a 5% margin of error, and a 50% response distribution, yielding a minimum of 386 participants. After excluding participants who did not match the inclusion criteria or provided inconsistent responses, 646 valid responses were obtained. Given the high but uncertain prevalence of sedentary behavior among females in previous studies, oversampling enhanced statistical power, reduced exclusions resulting from incomplete or inconsistent data, and enhanced the generalizability of results. Despite the initial OpenEpi calculation estimating a minimum of 386 individuals, a larger sample was recruited to ensure robust subgroup analyses and a comprehensive assessment of breathlessness and physical activity in women with sedentary behavior.

### 2.3. Data Collection Procedure

A snowball sampling strategy was used to improve participant recruitment and access to hard-to-reach population demographics. The survey was conducted online, and informed consent was sought prior to initiating the survey.

### 2.4. Instrument

An online self-administered questionnaire was distributed to participants. The first section included self-reported questions assessing sociodemographic characteristics such as age, height, weight, marital status, educational level, smoking history, psychological problems, employment, and chronic diseases. The investigators calculated BMI using the standard formula BMI = weight (kg)/height^2^ (m^2^) based on self-reported body weight and height provided by the participants. Patients with chronic illnesses, being those who self-reported having arthritis, diabetes mellitus, chronic lung disease (COPD or asthma), coronary artery disease, heart failure, hypertension, stroke, and peripheral arterial disease, were excluded. The second section included the following scales and questionnaire: Modified Medical Research Council breathlessness scale (mMRC) [24] and validated Arabic version of Dyspnoea-12 Questionnaire (D-12) [25]. The third part of the questionnaire included the validated Arabic version of the Godin Leisure-Time Exercise Questionnaire (GLTEQ) to evaluate the individuals’ level of physical activity [26].

The mMRC is a five-point ordinal scale (0–4) that correlates exertion level with breathlessness. Higher ratings indicate greater functional impairment because of breathlessness. The mMRC Breathlessness scale is a questionnaire which consists of five statements related experienced breathlessness: grade 0, “I only get breathless with strenuous exercise”; grade 1, “I get short of breath when hurrying on the level or up a slight hill”; grade 2, “I walk slower than people of the same age on the level because of breathlessness or have to stop for breath when walking at my own pace on the level”; grade 3, “I stop for breath after walking 100 yards or after a few minutes on the level”; and grade 4, “I am too breathless to leave the house” or “breathless when dressing”. To establish an Arabic version of the mMRC breathlessness scale, two expert translators translated the English version. An expert panel (two respiratory physicians, one physical therapist, and one respiratory therapist) then compared the two translations to the original to resolve any discrepancies and agreed on a single Arabic version. Then, two independent translators did a back-translation into English, which was compared to the original to ensure consistency. Five experts validated the questionnaire’s face and content validity. The Arabic version was pre-tested on 10 sedentary participants to check item clarity, then piloted in a group of 30 sedentary females to assess reliability. Cronbach’s alpha was 0.85, indicating high internal consistency. The D-12 is a 12-item Arabic questionnaire that includes dyspnea characteristics in two domains: physical (items 1–7) and affective (items 8–12). Each question has a score between 0 and 3, for a total score between 0–36.

The Godin Leisure-Time Exercise Questionnaire has been validated through comparisons with accelerometers [27,28], VO_2_max (maximum oxygen uptake), and body fat percentage [29,30]. The Godin Leisure-Time Exercise Questionnaire (GLTEQ) has the potential to address the challenges associated with commonly used physical activity self-report measures by minimizing participant and researcher burden and improving responsiveness [31]. Participants’ frequency of light, moderate, and strenuous physical activity over the seven days prior to the survey was reported. The questionnaire describes each of these three components and provides an example of their activities. Participants were advised to carefully read every section and report how many times they performed the activity per week. The GLTEQ leisure score index was computed based on how often they engaged in intense, moderate, or mild exercise. Every workout type’s number is further multiplied by a constant value for every activity type. The number of repetitions is multiplied by nine for intense exercise, five for moderate exercise, and three for mild exercise. For instance, if the participants reported having completed three sessions of intense exercise, two sessions of mild exercise, and four sessions of moderate exercise, the number of sessions of intense exercise was multiplied by 9 (3 × 9 = 27), the number of sessions of moderate exercise by 5 (4 × 5 = 20), and the number of mild exercise sessions by 3 (2 × 3 = 6). The GLTEQ leisure score index was determined by adding these three scores, which came out to be 27 + 20 + 6 = 53. Participants were classified as active if their GLTEQ leisure score index total scores were 24 or higher, as moderately active if they scored between 14 and 23, and insufficiently active if they scored less than 14.

### 2.5. Risk of Bias

The study’s dependence on self-administered questionnaires may include biases associated with self-reporting, such as recall bias or social desirability bias, especially for items evaluating physical activity and sedentary behavior. The utilization of a single item from the IPAQ-SF to evaluate sedentary behavior may restrict the depth of the assessment. While thorough translation and validation processes for the Arabic versions of the mMRC, there exists a possibility of cultural or language misinterpretation. The online-only survey methodology might exclude people lacking internet access, potentially compromising the sample’s representativeness. The snowball sampling method poses a risk of selection bias, since participants may selectively recruit individuals with identical characteristics.

### 2.6. Statistical Analysis

Descriptive and inferential statistical methods were employed to examine the associations between the 646 participants’ demographic characteristics, physical activity, and dyspnea. Not all numerical variables were distributed normally; hence, medians and interquartile ranges (IQR) were employed. Categorical variables were evaluated using frequency distributions and percentages. Non-parametric statistics were applied to account for the skewed distributions. A Kruskal–Wallis test was used to compare Total Dyspnea 12 scores and Modified Breathlessness Scale grades across three activity levels (Active, Moderately Active, and Insufficiently Active) based on GLTEQ leisure scores. To account for multiple comparisons, the *p*-values were adjusted with the Bonferroni correction technique, which aids in the control of the family-wise error rate. The significance threshold is set at *p* < 0.017 due to the Bonferroni adjustment divides the conventional alpha level (0.05) by the number of comparisons (3 in this case), resulting in more rigorous criteria for statistical significance. The ordinal logistic regression model was used to predict physical activity levels based on the predictors. To verify that the model was valid, the proportionate odds assumption was examined, and odds ratios (OR) with 95% CIs were computed to evaluate the strength of associations. Statistical significance was established at *p* < 0.05. All analyses were executed using R Statistical Software (version 4.1.2; R Core Team, 2021).

## 3. Results

### 3.1. Descriptive Statistics for Study Variables

Among the 646 participants, the median BMI was 24.0 kg/m^2^ (IQR: 21.3–27.9), and the median leisure-time physical activity score (GLTEQ) was 18.0 (IQR: 6.5–29.0). The median total Dyspnea-12 score was 3.0 (IQR: 1.0–8.0), with the Physical and Emotional Domain medians at 3.0 (IQR: 1.0–6.0) and 0.0 (IQR: 0.0–2.0), respectively. Approximately 37% of participants were insufficiently active, 35% active, and 28% moderately active. Most (95%) reported some degree of breathlessness, predominantly Grade 2 (32%) and Grade 3 (24%). The sample was composed mainly of individuals aged 20–30 years (60%) and students (36%), with the remainder being unemployed (35%) or employed (28%).

#### Differences in Dyspnea According to Activity Levels

The Kruskal–Wallis tests indicated statistically significant differences in Total Dyspnea 12 scores (H(2) = 50.43, *p* < 0.0001) and Modified Breathlessness Scale grades (H(2) = 320.45, *p* < 0.0001) among different levels of physical activity. Table 1 presents the results of a post hoc analysis conducted using Dunn’s test with Bonferroni correction. The findings indicate that Total Dyspnea 12 scores were significantly lower in the Active group when compared to both the Insufficiently Active group (corrected *p* = 0.0001) and the Moderately Active group (corrected *p* = 0.0001). No significant difference was observed between the Insufficiently Active and Moderately Active groups (corrected *p* = 1.000). Active individuals exhibit significantly lower levels of dyspnea compared to their less active counterparts, while the severity of dyspnea does not show a notable difference between Insufficiently Active and Moderately Active individuals. Regarding the Modified Breathlessness Scale, Table 1 shows that Dunn’s test identified significant differences between all pairs of activity levels (all corrected *p*-values = 0.0001). Participants in the Active group demonstrated the lowest levels of breathlessness, whereas those in the Insufficiently Active group exhibited the highest levels. The findings demonstrate that elevated breathlessness grades, especially grade 3, are more common among Insufficiently Active participants, underscoring a significant correlation between diminished physical activity and heightened severity of activity-related dyspnea.

### 3.2. Association Between Dyspnea Measures and Physical Activity Levels

As shown in Table 2, Ordinal logistic regression analysis examined the association between dyspnea measures and physical activity levels. Three predictors were analyzed: the modified Medical Research Council (mMRC) dyspnea score, the Dyspnea-12 physical domain, and the Dyspnea-12 emotional domain. Models were estimated with and without adjustments for age, BMI, occupation, and sitting time.

In the unadjusted model, a one-unit increase in mMRC score was associated with an 81% decrease in the odds of higher physical activity levels (OR = 0.19, 95% CI [0.16, 0.23], *p* < 0.001). This association remained significant in the adjusted model (OR = 0.21, 95% CI [0.17, 0.25], *p* < 0.001), indicating a strong inverse relationship between breathlessness severity and physical activity.

For the Dyspnea-12 physical domain, each additional unit was associated with a 7% decrease in the odds of higher physical activity (OR = 0.93, 95% CI [0.90, 0.96], *p* < 0.001). The association remained significant after adjustment, reinforcing the role of physical dyspnea symptoms in limiting activity levels. In contrast, the Dyspnea-12 emotional domain was not significantly associated with physical activity. The unadjusted model showed OR = 0.96 (95% CI [0.91, 1.01], *p* = 0.103), and the adjusted model remained non-significant (OR = 0.95, 95% CI [0.90, 1.00], *p* = 0.068). Among confounders, longer sitting time was significantly associated with lower activity (OR = 0.97, 95% CI: 0.94–1.00; *p* = 0.032), while unemployment was linked to reduced odds of higher activity (OR = 0.78, *p* = 0.041). BMI and age were not significantly associated with activity levels.

#### Model Fit Statistics

The mMRC model demonstrated the strongest fit (Pseudo R^2^ = 0.3075, Log-Likelihood = −1006.64, AIC = 2023.27, BIC = 2044.40), outperforming the Dyspnea-12 physical domain model (Pseudo R^2^ = 0.1125, Log-Likelihood = −1290.28, AIC = 2590.57) and the Dyspnea-12 emotional domain model, which had minimal explanatory power (Pseudo R^2^ = 0.0047, Log-Likelihood = −1445.37, AIC = 2900.74). These findings highlight that physical aspects of breathlessness, particularly were evaluated by the mMRC and Dyspnea-12 physical domains, are substantial and consistent predictors of reduced physical activity levels in women with sedentary behaviour.

## 4. Discussion

In this study of adult females in Saudi Arabia with sedentary behavior, significant levels of physical inactivity were observed, with 37.36% categorized as insufficiently active.

A significant prevalence of dyspnea was observed, with 95.2% of individuals reporting some level of breathlessness. The majority experienced moderate to severe breathlessness (Grade 2: 32.2%; Grade 3: 24.46%). This finding aligns with previous research by Bahathig et al. [32] who reported that 92.7% of Saudi female adolescents did not meet the recommended 420 min of moderate-to-intense physical activity per week. Furthermore, a 2020 national survey found that 91.1% of Saudi women were inactive [33]. This issue is influenced by multiple factors that disproportionately impact women in Saudi Arabia.

Socioeconomic development has resulted in lifestyle changes characterized by reduced physical activity and heightened sedentary behaviors, particularly among women in office-based positions [34]. Cultural expectations and common gender roles frequently emphasize domestic responsibilities at the expense of personal health and leisure activities, thereby restricting possibilities for physical engagement [35]. Environmental barriers, such as extreme heat, discourage outdoor exercise [7]. Additionally, restricted access to supportive facilities and time constraints further diminish activity levels [36]. Historically, women’s mobility was restricted by the previous driving ban and inadequate public transportation options, which further contributed to physical inactivity [37]. The interconnected factors could clarify the increased sedentary behavior and related symptoms, such as breathlessness, observed among women in this study.

The study also revealed a significant difference in dyspnea severity across activity levels, with insufficiently active individuals reporting higher Total Dyspnea-12 scores and Modified Breathlessness Scale grades compared to those who were active (*p* < 0.017). The findings indicate that higher dyspnea extent, as evaluated by the mMRC and Dyspnea-12 Physical Domain, was substantially associated with decreased physical activity levels, even after adjusting for confounders. Longer sitting times and unemployment were similarly linked to decreased exercise, although BMI and age had no significant association.

Dyspnea has been identified as a sign of poor cardiovascular fitness, particularly in the world’s increasingly sedentary populations [10]. Our findings are consistent with research by Vaz Fragoso et al. [14], who observed that moderate-to-severe dyspnea affected 31.6% of older, sedentary individuals, which was closely associated with decreased gait speed and increased sedentary time. These findings emphasize the role of respiratory impairment and dyspnea as predictors of physical inactivity in older sedentary populations, aligning with the patterns observed in our study.

Carballo-Fazanes et al. [38] similarly found that increased physical activity was associated with decreased sedentary behavior, better respiratory function, and less breathlessness. In addition, previous studies have shown that among healthy young individuals, better pulmonary function is associated with greater 6-min walk distance and higher levels of physical activity, as assessed using the International Physical Activity Questionnaire (IPAQ) categories [6]. Furthermore, a study evaluating the effects of sedentary behavior and moderate-to-vigorous physical activity on pulmonary function among healthy participants found that increased sedentary behavior negatively affected pulmonary function, particularly reducing FEV1 and FVC. Although regular moderate-to-vigorous physical activity was found to reduce these negative effects, its benefits were restricted for those sitting more than 8 h per day [39].

Dyspnea measures play a wider role in predicting health-related quality of life and survival outcomes because they offer distinct insights beyond what can be captured by pulmonary function tests [40]. Previous studies have suggested that dyspnea is a complex phenomenon consisting of various sensations rather than a single perception [10,41].

Although understanding of the mechanisms underlying dyspnea has advanced, little is known about the variability of dyspnea perception in healthy subjects, whether at rest or during exertion [42]. This variation may influence how individuals with dyspnea approach physical activity. In healthy individuals, spontaneous breathing is typically unobstructed, and the respiratory system functions harmoniously, so they tend to avoid unpleasant sensations associated with breathing [43]. However, experimental procedures, including mechanical and chemical stress of the respiratory system, can elicit unpleasant respiratory symptoms in otherwise healthy individuals [44,45,46,47,48]. For instance, restricting tidal volume (VT) during spontaneous breathing in the presence of enhanced or continuous chemostimulation can provoke a sensation of “air hunger” [49]. Similarly, when healthy young individuals exercise, the combination of external mechanical loading (like limited chest wall expansion) and chemical intervention (like adding more dead space equivalent to CO_2_ rebreathing) substantially raises the severity of breathing discomfort and increases exercise intolerance [50]. This evidence reinforces that decreased activity levels in individuals with dyspnea can be attributed to both subjective respiratory discomfort and physical limitations.

Several studies have attempted to explain the association between dyspnea and decreased physical activity level. O’Donnell et al. [51] explained that many individuals choose to live sedentary lifestyles in order to avoid exertional dyspnea, which can result in severe skeletal muscle deconditioning, poor psychological outcomes, and social isolation. In addition to reducing physical activity, chronic dyspnea is associated with increased mortality rates and diminished health-related quality of life. Dyspnea significantly impairs physical functioning, and effectively managing it requires an understanding of its complex mechanisms [51].

In sedentary people, exertional dyspnea is frequently experienced, especially among older adults. Shortness of breath is a common complaint during simple physical activities like walking or climbing stairs. Prolonged inactivity can worsen this symptom, which is frequently linked to decreased ventilatory capacity and respiratory muscle weakness [14]. Extended periods of sedentary behavior significantly impair cardiorespiratory fitness, as demonstrated by rapid reductions in maximal oxygen uptake ($\dot{V}$O_2_max). A 14-day decreased daily steps resulted in a 6.6% reduction in $\dot{V}$O_2_max among healthy adults, with this decline occurring at a significantly accelerated rate compared to the age-related reduction in $\dot{V}$O_2_max, which averages approximately 10% per decade, irrespective of physical activity levels, in both females and males. The duration of time spent in sedentary behavior was negatively correlated with changes in $\dot{V}$O_2_max, independent of the time allocated to moderate-to-vigorous physical activity [52].

Sedentary behavior accelerates muscle deconditioning, leading to a rapid decline in skeletal muscle mass and strength. Significant reductions in muscle mass have been consistently observed after just a few days of increased sedentary behavior, regardless of the study model used. Periods of immobilization or reduced physical activity lasting 5 to 14 days can result in substantial decreases in lower limb muscle mass up to 9% and strength losses ranging from 8% to 25% [53,54,55,56]. Additionally, prolonged inactivity shifts muscle fiber composition toward fast-twitch fibers and reduces oxidative capacity, increasing fatigue and reliance on anaerobic metabolism [57,58], thereby exacerbating dyspnea symptoms.

The practical implications of the study underscore the association between breathlessness and low levels of physical activity, emphasizing the necessity of strategies that address both. Pulmonary rehabilitation programs involving breathing exercises, physical training, and psychological support may help people manage their breathlessness while exercising. Furthermore, objective measures of physical activity, such as accelerometry, can assist clinicians in better understanding and addressing the activity restrictions associated with breathlessness. Finally, customized therapies and public health efforts that encourage tolerable physical activity can interrupt the cycle of inactivity, sedentary lifestyle, and improve overall health outcomes for people suffering from dyspnea.

## 5. Strengths and Limitations

This study has significant strengths, particularly its focus on women with sedentary behavior, a demographic that is frequently overlooked in physical activity studies, which increases the significance of the findings for targeted health treatments. This research has some limitations. The use of convenience and snowball sampling introduces selection bias, which may limit the data’ generalizability to a larger sedentary female group. Furthermore, the reliance on online surveys limits participation to those with internet access and digital literacy, thus excluding crucial sectors of the target audience. Furthermore, the reliance on self-reported measures of physical activity and sedentary behavior may be influenced by recollection bias and social desirability bias, thus compromising data accuracy. Despite extensive translation and cross-cultural adaptation, linguistic and cultural sensitivities in the Arabic version of the mMRC breathlessness scale may have influenced participant responses, resulting in inconsistencies in interpretation. Furthermore, the cross-sectional design limits the ability to determine the causal relationship between activity-related dyspnea and physical activity levels. Future investigations utilizing more representative sample techniques and longitudinal designs are recommended to validate and extend these results.

## 6. Conclusions and Future Recommendations

This study observed a high prevalence of activity-related dyspnea in women with sedentary behaviour, emphasizing a significant health issue within this population. Moreover, increased severity of dyspnea correlated significantly with reduced levels of physical activity. The findings underscore the importance of early identification and management of breathlessness to enhance participation in physical activity and improve overall health outcomes. Public health initiatives should prioritize the development and implementation of tailored physical activity programs that target the barriers encountered by sedentary women, especially those suffering from breathlessness.

Future research should employ longitudinal designs to further examine the physiological, psychological, and inflammatory mechanisms that contribute to dyspnea and inactivity. Incorporating diverse demographic groups, including men and older adults, would improve the understanding of these relationships. Clinical trials assessing targeted interventions, including breathing exercises and graded exercise programs, will be essential for identifying effective strategies to enhance physical activity and alleviate dyspnea in this population. The implementation of evidence-based interventions may significantly improve public health outcomes for populations at risk due to sedentary lifestyles.

## Figures and Tables

**Table 1:** Dunn’s Test Results for Total Dyspnea 12 Score and Modified Breathlessness Scale.

**Dunn’s Test Results for Total Dyspnea 12 Score**				
**Group 1**	**Group 2**	**Mann-Whitney U**	***p*** **-Value**	**Corrected *p*-Value**
Active	Insufficiently active	17,963.0	0.0001	0.0001 *
Active	Moderately active	13,212.5	0.0001	0.0001 *
Insufficiently active	Moderately active	21,313.5	0.687	1.000
**Dunn’s Test Results for Modified Breathlessness Scale**				
**Group 1**	**Group 2**	**Mann-Whitney U**	***p*** **-Value**	**Corrected *p*-Value**
Active	Insufficiently active	4039.5	0.0001	0.0001 *
Active	Moderately active	5645.5	0.0001	0.0001 *
Insufficiently active	Moderately active	29,740.5	0.0001	0.0001 *

* Statistically significant after Bonferroni correction (corrected *p* < 0.017).

**Table 2:** Ordinal Logistic Regression Results Predicting Physical Activity Levels.

Variable	Unadjusted OR (95% CI)	*p*-Value	Adjusted OR (95% CI)	*p*-Value	Pseudo R-Squared
mMRC Score	0.19 (0.16–0.23)	<0.001	0.21 (0.17–0.25)	<0.001	0.3075
Dyspnea-12 Physical Domain	0.92 (0.89–0.95)	<0.001	0.93 (0.90–0.96)	<0.001	0.1125
Dyspnea-12 Emotional Domain	0.96 (0.91–1.01)	0.1030	0.95 (0.90–1.00)	0.0680	0.0047
Model Fit Statistics:					
Model	Log-Likelihood	AIC	BIC		
mMRC Score (0–4)	−1006.64	2023.27	2044.4		
Dyspnea-12 Physical Domain	−1290.28	2590.57	2611.69		
Dyspnea-12 Emotional Domain	−1445.37	2900.74	2921.87		
Confounder Results (Adjusted Model Only):					
Confounder	OR (95% CI)	*p*-Value			
BMI	0.98 (0.95–1.01)	0.1542			
Sitting Time	0.97 (0.94–1.00)	0.0321			
Age (31–40 vs. 21–30)	1.25 (0.98–1.59)	0.0723			
Age (41–50 vs. 21–30)	0.89 (0.70–1.13)	0.3421			
Occupation (Student vs. Employed)	1.12 (0.88–1.42)	0.3651			
Occupation (Unemployed vs. Employed)	0.78 (0.61–0.99)	0.0412			

mMRC = modified Medical Research Council dyspnea scale; OR = Odds Ratio; CI = Confidence Interval; *p*-values < 0.05 are considered statistically significant; Pseudo R-squared values range from 0 to 1, with higher values indicating better model fit; AIC = Akaike Information Criterion; BIC = Bayesian Information Criterion (lower values indicate better model fit); Adjusted models include Age, BMI, Occupation, and Sitting Time as covariates.

## Data Availability

Data used in this study can be obtained upon reasonable request from the corresponding author.

## Abbreviations

The following abbreviations are used in this manuscript:

D-12	Dyspnea-12
GLTEQ	Godin Leisure-Time Exercise Questionnaire
mMRC	Modified Medical Research Council breathlessness scale
